# Ramipril can alleviate the accumulation of renal mesangial matrix in rats with diabetic nephropathy by inhibiting insulin-like growth factor-1^1^

**DOI:** 10.1590/s0102-865020190010000007

**Published:** 2019-02-14

**Authors:** Wei Ren, Chen Zhao, Yan Wang, Yuan Fang, Zhenzhen Huang, Wei Chen, Lihua Wang, Wen Hu, Ke Wang, Lijun Ni

**Affiliations:** I Master, Department of Nephrology, Anhui Provincial Hospital Affiliated to Anhui Medical University, Hefei 230001, China. Intellectual, scientific, conception and design of the study; acquisition, analysis and interpretation of data; technical procedures; statistical analysis; manuscript preparation; final approval.; II MD, Department of Nephrology, Anhui Provincial Hospital Affiliated to Anhui Medical University, Hefei 230001, China. Acquisition, analysis and interpretation of data; technical procedures; statistical analysis; manuscript writing.; III Master, Department of Nephrology, Anhui Provincial Hospital Affiliated to Anhui Medical University, Hefei 230001, China. Acquisition of data, technical procedures.; IV MD, Department of Nephrology, Anhui Provincial Hospital Affiliated to Anhui Medical University, Hefei 230001, China. Acquisition of data, technical procedures.; V MD, Department of Nephrology, Anhui Provincial Hospital Affiliated to Anhui Medical University, Hefei 230001, China. Acquisition of data.; VI Master, Department of Nephrology, Anhui Provincial Hospital Affiliated to Anhui Medical University, Hefei 230001, China. Acquisition of data.; VII Master, Department of Pathology, Anhui Provincial Hospital Affiliated to Anhui Medical University, Hefei 230001, China. Acquisition, analysis and interpretation of data; technical procedures.

**Keywords:** Ramipril, Insulin-Like Growth Factor I, Diabetic Nephropathies, Rats

## Abstract

**Purpose:**

To investigate the impact of Ramipril (RAM) on the expressions of insulin-like growth factor-1 (IGF-1) and renal mesangial matrix (RMM) in rats with diabetic nephropathy (DN).

**Methods:**

The Sprague Dawley rats were divided into normal control (NC) group (n = 12), DN group (n = 11), and DN+RAM group (n = 12). The ratio of renal weight to body weight (RBT), fasting blood glucose (FBG), HbA1c, 24-h urine protein (TPU), blood urea nitrogen (BUN), creatinine (Cr), renal pathological changes, the levels of IGF-1, fibronectin (FN), type IV collagen (Col-IV), and matrix metalloproteinases (MMP)-2 were compared among the groups.

**Results:**

Compared with NC group, the RBT, FBG, HbA1c, TPU, BUN, Cr, and RMM in DN group were significantly increased (P < 0.05), the IGF-1, FN, and Col-IV were significantly upregulated (P < 0.05), while MMP was significantly downregulated (P < 0.05). Compared with DN group, the indexes except for the FBG and HbA1c in DN+RAM group were significantly improved (P < 0.05), among which IGF-1 exhibited significant positive correlation with TPU(r=0.937), FN(r=0.896) and Col-IV(r=0.871), while significant negative correlation with MMP-2 (r=-0.826) (P<0.05).

**Conclusion:**

RAM may protect the kidneys by suppressing IGF-1 and mitigating the accumulation of RMM.

## Introduction

 Diabetes mellitus (DM) is a chronic metabolic disease severely threatening the public’s health. It is involved in many organs of the body with high mortality and morbidity. One epidemiological investigation has shown that the prevalence of DM in Chinese adults in 2013 was increased to 11.6%, and the total number had reached 114 million^1^ ^,^ ^2^. Diabetic nephropathy (DN) is a common chronic complication of DM as well as the leading cause of end stage renal failure (ESRD) and death^3^ ^,^ ^4^. The characteristic pathological changes of DN are glomerular mesangial cell hypertrophy, basement membrane thickening, and excessive accumulation of extracellular matrix, which thus cause progressive glomerular sclerosis. The pathogenesis is complex and has not been fully elucidated yet^5^ ^,^ ^6^. Existing studies have shown that the accumulation of mesangial extracellular matrix is one of the most prominent pathological changes in DN, and it’s also the important pathological basis for nodular or diffuse glomerulosclerosis and the occurrence and development of DN^7^ ^,^ ^8^. Therefore, exploring the mechanism of mesangial extracellular matrix accumulation and new therapeutic targets have become hot research spots.

 Insulin-like growth factor-1 (IGF-1) is a single-chain growth factor composed of 70 amino acids, and has similar structure and functions to insulin such as promoting cell differentiation and proliferation, and exhibiting insulin-like metabolic roles^9^. Levin-Iaina *et al*.^10^ reported the renal IGF-1 protein is significantly upregulated in rats with Streptozotocin (STZ)-induced early DM, which is also related to renal compensatory hypertrophy and high filtration; Li *et al*.^11^ found the serum IGF-1 level is significantly increased in patients with type 2 diabetic nephropathy, which also increases the progress of DN, thus suggesting that IGF-1 may be closely related to the occurrence and development of DN^12^. Previous studies have shown that angiotensin converting-enzyme inhibitors (ACEI) can improve the synthesis of mesangial matrix, reduce proteinuria in DN patients, and delay disease progression, so it has become a guide drug for the treatment of DN^13^. However, studies about the roles of IGF-1are rare in China and abroad. This study observed the effects of ramipril (RAM) on the expression changes of IGF-1, fibronectin (FN), type IV collagen (Col-IV), and matrix metalloproteinases (MMP)-2 in STZ-induced DN rats, hoping to explore the possible mechanism of ACEI in treating DN.

## Methods

 This study was carried out in strict accordance with the recommendations in the Guide for the Care and Use of Laboratory Animals of the National Institutes of Health. The animal use protocol has been reviewed and approved by the Institutional Animal Care and Use Committee (IACUC) of Anhui Medical University. 

 A total of 36 healthy SPF-grade 8-week-old male Sprague Dawley rats, body weight 190 ± 10g, were provided by the Experimental Center of Anhui Medical University (Certificate No. SCXK (Wan) 2005-001) and bred with free diet and water for one-week adaption. This study was conducted in the central laboratory of Anhui Provincial Hospital Affiliated to Anhui Medical University.

 The rats were then divided into group normal control (NC, n = 12), group DN (n = 12), and group DN+RAM (n = 12) using the random number method. The rats in group DN and DN+RAM were intraperitoneally injected 60 mg/kg STZ (Sigma-Aldrich, St. Louis, USA) after 14-h fasting, and sampled the venous blood from the tail vein 48 h, 72 h, 1 w, 2 w, and 4 w later, together with the urine samples. The signs of blood glucose >16.7 mmol/L and urinary protein excretion rate > 30 mg/24 h can be seen as the successful establishment of the DN rat model^14^. In group DN, one rat was excluded due to not achieving the blood glucose standard. Group NC was intraperitoneally injected the equal volume of citrate buffer. After succeeding in model preparation, group DN+RAM was orally administrated 3 mg/kg/d RAM (Sanofi-Aventis, Paris, France, 2.5 mg/tablet, approval number: H20040517) for 8 weeks , while group NC and DN were given the equal volume of distilled water. During the experiment, standard diet and free water were supplied while no insulin was applied.

###  Specimen collection 

 Each rat was collected 24-h urine at the end of the 8th week, after which the rat was killed by intraperitoneally injecting 10% chloral hydrate, sampled t 4-5 mL of cardiac blood for detecting the biochemical indicators in the serum. The left kidney was weighed so as to calculate the ratio of renal weight/body weight (mg/g); partial renal tissue was then fixed in 10% neutral formalin and embedded by paraffin; the rest was cut on ice and stored at -80 °C.

###  Determination of biochemical indicators 

 The fasting blood glucose (FBG), blood urea nitrogen (BUN) and serum creatinine (Cr) were determined using one Hitachi 7600-020 automatic biochemical analyzer (Hitachi, Ltd., Tokyo, Japan). The HbA_1c_ values were detected by the HPLC method (VARIANT II Hemoglobin A_1c_ Testing System, BIO-RAD Corp., Hercules, CA, USA) and the 24-hours proteinuria (TPU) was measured by immunoturbidimetry. All biochemical indicators were assayed in the biochemical laboratory of Anhui Provincial Hospital Affiliated to Anhui Medical University.

###  Renal pathology 

 The paraffin sections were prepared consecutive slices with 2-3 mm in thickness, followed by conventional dewaxing, HE staining, and observing the histopathological changes under one light microscope (400 times). Each image was taken randomly 10 different visual fields. Partial renal cortex was cut into l mm^3^ cubes, fixed with 2.5% glutaraldehyde, prepared the ultra-thin slices, preformed Pb-U staining, and observed the renal pathological changes using one JEM-1230 transmission electron microscope (TEM, JEOL Ltd., Tokyo, Japan).

###  Immunohistochemistry 

 The expression of IGF-1 in the renal tissue was detected by the Elivision method: The paraffin sections were prepared consecutive slices with 4 mm in thickness, followed by dewaxing, hydration, 3% H_2_O_2_ blocking (to eliminate the activity of endogenous peroxidase), PBS washing, and overnight incubation with 50 μl of rabbit anti-rat IGF-1 antibody (Boster Ltd., Wuhan, China) at 4°C (PBS was used to replace the primary antibody as the negative control). The slices were then added the polymer enhancer and the enzyme-labeled goat anti-rabbit polymer (Maxim Bioengineering Ltd. Fuzhou, China) drop wisely for 20-min culture at 37°C, followed by drop wisely adding DAB reagent and microscopically controlled coloration, hematoxylin re-staining, dehydrating, hyalinization, and gum mounting. 20 visual fields in the cortex region were randomly selected under light microscope (×200) and analyzed the accumulative optical density and total area of IGF-1 positive signal by Image Pro Plus 6.0; the average optical density (AOD) of the positive signal was then calculated.

###  Western blot 

 100 mg of cryopreserved renal tissue was added 1 ml of total protein extract solution for 30-min ice lysis, followed by 15-min centrifugation at 4°C and 12000 r/min, and the supernatant was then taken to determine the total protein concentration by BCA. 60 μg of the protein was electrophoresed by SDS-PAGE and then transferred onto one PVDF membrane at 260 mA for 90 min; the membrane was then blocked with 5% nonfat dry milk for 1 h, added rabbit anti-rat IGF-1, FN, Col-IV, and MMP-2 monoclonal antibodies (diluted 1: 1000, Santa Cruz, CA, USA), respectively, and incubated overnight at 4°C. After washed with TBST, the membrane was added the horseradish peroxidase (HRP) labeled secondary antibody, incubated at room temperature after 1 h, and performed enhanced chemiluminecence (ECL) coloration. The bands were analyzed by Quantity One software, and the relative expressions of the target proteins were expressed referring to β-actin in the same specimen.

###  Statistical analysis 

 SPSS 19.0 was used for the statistical analysis; the measurement data were expressed as ±s; the multi-group comparison used the single factor analysis of variance, and comparison between two groups used the SNK test; the correlation analysis used the Pearson linear correlation analysis, with P <0.05 considered as statistically significance.

## Results

###  General conditions and biochemical indexes 

 Compared with group NC, the rats in group DN appeared such obvious symptoms as polydipsia, polyphagia, and polyuria, as well as poor spirit and activity, after 8-week medication. The symptoms in group DN+RAM were improved than group DN. Compared with group NC, the RBT (6.31±0.85 *vs.* 2.76±0.24 mg/g), FBG (24.53±1.21 *vs.* 5.07±0.38 mmol/L), HbA1c (11.46±0.67 *vs.* 4.72±0.55%), TPU (120.52±24.39 *vs.* 3.49±1.16 mg), BUN (19.39±2.07 *vs.* 7.84±0.61), Cr (97.82±8.65 *vs.* 34.96±5.17 μmol/L) in group DN were significantly increased (P <0.05). Compared with group DN, there was no significant difference in other indexes except for FBG (23.96±2.03 *vs.* 24.53±1.21 mmol/L) and HbA1c (11.28±0.81 *vs.* 11.46±0.67%) in group DN+RAM (P> 0.05) (Table 1).

**Table 1:** Comparison of general conditions and biochemical indexes among the three groups at the 8 week (±s).

Group	n	RBT (mg/g)	FBG(mmol/L)	HbA1c(%)	TPU(mg)	BUN(mmol/L)	Cr(μmol/L)
NC	12	2.76±0.24	5.07±0.38	4.72±0.55	3.49±1.16	7.84±0.61	34.96±5.17
DN	11	6.31±0.85^#^	24.53±1.21^#^	11.46±0.67^#^	120.52±24.39^#^	19.39±2.07^#^	97.82±8.65^#^
DN+RAM	12	5.28±0.73^#,&^	23.96±2.03^#^	11.28±0.81^#^	56.48±12.77^#,&^	11.42±1.45^#,&^	53.24±6.49^#,&^

Note: compared with group NC,^#^ *P*<0.05; compared with group DN,^&^ *P*<0.05.

 The RBT (6.31±0.85 *vs.* 2.76±0.24 mg/g), FBG (24.53±1.21 *vs.* 5.07±0.38 mmol/L), HbA1c (11.46±0.67 *vs.* 4.72±0.55%), TPU (120.52±24.39 *vs.* 3.49±1.16 mg), BUN (19.39±2.07 *vs.* 7.84±0.61), Cr (97.82±8.65 *vs.* 34.96±5.17 μmol/L), and RMM in group DN were significantly increased, the IGF-1 (4.53±0.16 *vs.* 2.17±0.10), FN (3.87±0.19 *vs.* 2.56±0.15), and Col-IV (3.46±0.20 *vs.* 2.23±0.25) were significantly upregulated, while MMP (2.20±0.19 *vs.* 3.41±0.18) was significantly downregulated. Compared with group DN, the indexes except for the FBG (23.96±2.03 *vs.* 24.53±1.21 mmol/L) and HbA1c (11.28±0.81 *vs.* 11.46±0.67%) in group DN+RAM were significantly improved, among which IGF-1 and TPU (r=0.937), as well as FN (r=0.896) and Col-IV (r=0.871), exhibited significant positive correlation, while significant negative correlation with MMP-2 (r=-0.826, P<0.05).

###  Renal histopathological changes 

#### Light microscopy (×400)

**Figure 1:**
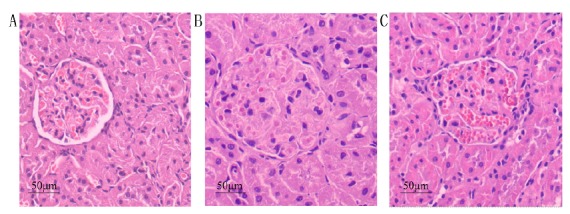
Pathological changes of renal tissue at the end of the 8th week (HE staining, ×400). **A**: NC; **B**: DN; **C**: DN+RAM.

 The glomerular appearance and structure in group NC showed no significant change, while compares with group NC, group DN exhibited glomerular hypertrophy, mesangial cell proliferation, significantly increased extracellular matrix, and significantly enlarged mesangial region. Compared with group DN, group DN+RAM exhibited significant improvement of the above pathological changes, the renal glomerulus reduce, the glomerular mesangial cells reduced, and the extracellular matrix only exhibited mild hyperplasia (Fig. 1).

#### TEM

**Figure 2:**
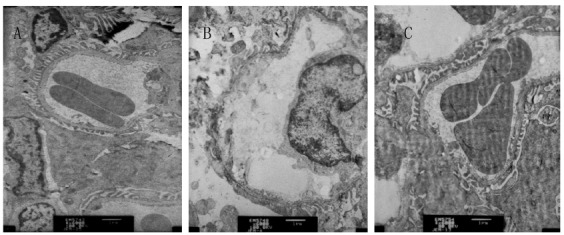
Changes of renal tissue of the three groups by TEM (Pb-U staining, ×6000). **A**: NC; **B**: DN; **C**: DN+RAM.

 Compared with group NC, the glomerular basement membrane in group DN was thickened, the mesangial matrix was proliferated and swollen, together with the deposition of a little electron dense material. Compared with group DN, the glomerular basement membrane in group DN+RAM showed no obvious thickening, and the mesangial matrix appeared mild hyperplasia (Fig. 2).

###  Expressions of IGF-1 by immunohistochemistry 

#### Immunohistochemisty

**Figure 3:**
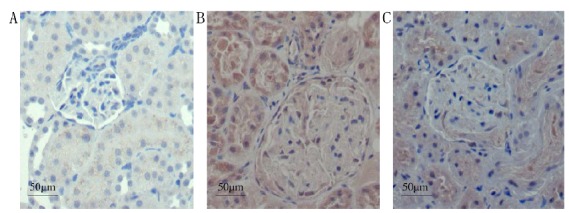
Immunohistochemisty of IGF-1in renal tissue of the three groups (Elivision, ×400). **A**: NC; **B**: DN; **C**: DN+RAM.

 The expression of IGF-1 in group NC was minor, but that in group DN was significantly increased, exhibiting staining in the cytoplasm and nuclei, mainly in the glomerular mesangial region, as well as in the renal tubules, with the AOD value as 0.37 ± 0.02. Group DN+RAM exhibited reduced staining in the glomerular mesangial region, with the AOD value as 0.24 ± 0.03, significantly lower than group DN (P <0.05), but still significantly higher than group NC (P <0.05) (Fig. 3).

###  Expressions of IGF-1, FN, Col-IV, and MMP-2 by western blot 

#### Western blot

 Compared with group NC, the proteins of IGF-1 (4.53±0.16 *vs.* 2.17±0.10), FN (3.87±0.19 *vs.* 2.56±0.15), and Col-IV (3.46±0.20 *vs.* 2.23±0.25) were significantly increased in group DN, while the MMP-2 protein (2.20±0.19 *vs.* 3.41±0.18) was significantly decreased (P <0.05). Compared with group DN, the IGF-1 (3.24±0.15 *vs.* 4.53±0.16), FN (3.11±0.12 *vs.* 3.87±0.19) and Col-IV (2.98±0.19 *vs.* 3.46±0.20) proteins in group DN+RAM were significantly downregulated, while the MMP-2 protein (2.83±0.14 *vs.* 2.20±0.19) was significantly upregulated (P <0.05) (Table 2).

**Table 2:** Comparison of relative expressions of FN, Col-IV, MMP-2, and IGF-1 proteins among the three groups (±s).

Group	n	FN	Col-IV	MMP-2	IGF-1
NC	12	2.56±0.15	2.23±0.25	3.41±0.18	2.17±0.10
DN	11	3.87±0.19^#^	3.46±0.20^#^	2.20±0.19^#^	4.53±0.16^#^
DN+RAM	12	3.11±0.12^#,&^	2.98±0.19^#,&^	2.83±0.14^#,&^	3.24±0.15^#,&^

Note: compared with group NC,^#^ *P*<0.05; compared with group DN, ^&^ *P*<0.05.

###  Correlation analysis 

 The Pearson correlation analysis showed that the expression of IGF-1 protein was significantly positively correlated with TPU (r = 0.937), FN (r = 0.896), and Col-IV (r = 0.871), while significantly negatively correlated with MMP-2 (r = -0.826) (P <0.05).

## Discussion

 The pathogenesis of DN is complex and may be related to gene, glucose metabolic disorder, hemodynamic disturbance, insulin resistance, oxidative stress, or immunoinflammatory reaction, and it has been a hot spot all over the world. The main pathological features of DN are glomerular mesangial cell hypertrophy, basement membrane thickening, mesangial cell proliferation, and excessive accumulation of extracellular matrix, as well as glomerular and renal tubulointerstitial fibrosis in late stages, which eventually leads to renal failure. The excessive accumulation of extracellular matrix in the mesangial area is the most important pathologic feature of DN, and it is the common result of the increase of FN, Col-IV, and laminin (LN) and the decrease of MMP-2^6^. MMP-2 is a zinc-dependent matrix metalloproteinase, as the main gelatinase that degrades FN, Col-IV, and LN in the mesangium, it can reduce the accumulation of extracellular matrix and plays an important role in the occurrence and development of DN^15^ ^,^ ^16^.

 The Western blot results in this study showed that the MMP-2 protein in group DN was significantly reduced, while the FN and Col-IV proteins were significantly increased. The immunohistochemical and Western blot results showed that the IGF-1 and IGF-1 proteins in group DN were significantly increased, negatively correlated with MMP-2, while positively correlated with TPU, FN, and Col-IV, suggesting that IGF-1 may downregulate MMP-2, which reduces its roles of degrading the FN and Col-IV proteins, thus increasing the deposition of mesangial matrix and resulting in the increase of TPU. After 8 weeks of treatment with RAM, the RBT, TPU, BUN, Cr, and the protein expressions of FN, Col-IV, and IGF-1 were significantly reduced, while MMP-2 was significantly increased. The pathological results also showed that group DN exhibited more serious renal hypertrophy and the accumulation of a large number of mesangial matrix, which were improved after applied RAM, suggesting that RAM may improve the accumulation of mesangial matrix and play a renal protective role by inhibiting the expression of IGF-1 protein.

 IGF-1 is a single-chain protein encoded by chromosome 12, consisting of 70 amino acids and with a molecular weight of about 7500; it has nearly 50% of structural homology with insulin^9^. IGF-1 is synthesized and secreted by the liver, kidneys, bones, and fat, and distributed widely in various tissues. It binds to specific IGF-1 receptors by autocrine or paracrine, thus promoting the cell proliferation and differentiation, inhibiting the apoptosis, promoting the protein synthesis, etc.; in addition, it has a certain cross-role with insulin receptors^9^. Kong *et al.* ^17^ found that insulin deficiency can upregulate the expressions of IGF-1 and its receptor IGF-1R in the renal mesangial cells and DN rats’ kidneys, thus causing mesangial cell proliferation as well as the increase of serum creatinine and urinary proteins; Singh *et al.* ^18^ found through proteomics that the overexpression of IGF-1 can activate the Akt/GSK-3β signal pathway, thus promoting the growth of mesangial cells and protein synthesis in DN. Many studies suggest that the overexpression of IGF-1 is an important risk factor for DN progression. This study further confirmed the role of IGF-1 in mesangial matrix aggregation in DN and suggest that it may be related to the expression inhibition of MMP-2.

 A large number of studies have confirmed that ACEI drugs can reduce the activity of the renin-angiotensin-aldosterone system, thereby improving the renal hemodynamics and playing a protective effect^13^. Recent studies have shown that ACEIs can play roles through non-hemodynamic factors, such as reducing inflammatory responses, reducing oxidative stress, inhibiting matrix protein synthesis, etc^19^ ^,^ ^20^. McLennan found that perindopril can inhibit the degradation of mesangial matrix in DN rats by upregulating MMP-2^21^. The results of this study were consistent with previous studies, and further suggest that the specific mechanism may be through inhibiting the expression of IGF-1. It is noticeable that this study didn’t find RAM can significantly improve the FBG and HbA1c in DN rats, suggesting that the expression of IGF-1 is independent from the changes of blood glucose.

 In short, RAM may inhibit the expression of IGF-1 in the renal tissue of DN rats, and upregulate the expression of MMP-2, thus reducing the accumulation of mesangial matrix and delaying the progression of DN, which may exhibit significance for clinical treatments.
